# Constructing Li-O-Vacancy Configuration Coupling with a Layered/Spinel Mixed Structure in Li-Deficient Li-Rich Layered Oxides to Realize Stable Oxygen Redox

**DOI:** 10.3390/ma19061240

**Published:** 2026-03-21

**Authors:** Yibin Zhang, Meng Wang, Bao Qiu, Zhaoping Liu

**Affiliations:** 1 Ningbo Institute of Materials Technology & Engineering (NIMTE), Chinese Academy of Sciences, Ningbo 315201, China; 2 Center of Materials Science and Optoelectronics Engineering, University of Chinese Academy of Sciences (UCAS), Beijing 100049, China

**Keywords:** Li-rich materials, Li-deficient, oxygen redox, Li-O-V configuration, spinel-like, Coulombic efficiency

## Abstract

**Highlights:**

- A Li-O-vacancy configuration is constructed in Li-rich layered oxides.
- Advanced STEM-iDPC is used to visualize structures.
- Anions achieve stable oxidation–reduction.
- The layered/spinel-like composite structure achieved superior structural and electrochemical stability.
- Ultra-high Coulombic efficiency is obtained during initial cycling.

**What are the main findings?**

- We visualized the Li-O-V configuration in lithium-rich layered oxides.
- We have verified the stability of the spinel/layered mixed structure.
- We have demonstrated that the Li-O-V configuration can achieve available anionic capacity.

**What are the implications of the main findings?**

- They provide structural design strategy for achieving stable lattice oxygen capacity.
- They develop a lithium-rich material that can achieve high initial Coulombic efficiency.
- They provide ideas for designing lithium defect materials.

**Abstract:**

Owing to the synergistic effect of cationic and anionic charge compensation, Li-rich layered oxide cathodes stand as the most promising candidates for next-generation high-energy-density Li-ion batteries. However, the unstable oxygen redox process triggers irreversible oxygen release and structural degradation of the layered framework, which further destabilizes the Li-O-Li configuration and leads to severe performance decay. In this work, a layered/spinel heterostructure coupled with a stabilized Li-O-vacancy configuration is successfully constructed in a Li-rich layered oxide cathode. This design enables outstanding structural and electrochemical stability, delivering an initial discharge capacity of 232 mAh g^−1^ with a Coulombic efficiency of 90.5%. Moreover, the cathode retains 86.5% of its capacity after 100 cycles. The proposed structural design strategy offers a new pathway toward high-performance Li-rich layered oxide cathodes.

## 1. Introduction

Rechargeable lithium-ion batteries (LIBs) are powering the gradual transition from traditional fuel-powered vehicles to electric vehicles. As the global market share of electric and hybrid electric vehicles continues to grow, there is a strong demand for low-cost, high-energy-density cathode materials. Compared with traditional layered NCM cathodes that rely solely on transition metal oxidation–reduction [1,2], in Li-rich layered oxides, lithium extraction is initially compensated by cationic oxidation, followed by anionic redox above ~4.45 V vs. Li^+^/Li. This additional anionic charge compensation enables discharge capacities exceeding 250 mAh·g^−1^ [3,4]. Li-rich layered oxides can be seen as a composite of LiTMO_2_ (TM = Ni, Co, and Mn) and Li_2_MnO_3_, representing one of the most promising cathode candidates for next-generation high-energy-density Li-ion batteries [5,6]. These materials are characterized by a honeycomb-ordered arrangement of Li and transition metals within the transition metal layer, often referred to as a “LiMn_6_ superstructure” [7,8]. The presence of reactive Li-O-Li configurations facilitates the generation of electron holes in the O 2p band, thereby contributing to the oxygen redox activity in Li-rich cathodes [9]. However, the instability of anionic redox is typically accompanied by undesirable oxygen release, which, in turn, leads to structural degradation, electrochemical performance decay, and electrolyte consumption [10,11,12].

The transformation from the conventional Li-O-TM configuration (each O^2−^ ion is coordinated with three Li^+^ and three TM ions in traditional layered NCM cathodes) to the engineered Li-O-Li configuration (O^2−^ coordinates with four Li^+^ and two TM ions in Li-rich NCM cathodes), which enables a substantial increase in capacity. However, the honeycomb-ordered superstructure fails to provide a stable framework for oxidized oxygen species and tends to induce extensive transition metal migration [13,14,15]. Although surface coating and lattice doping have been demonstrated to partially stabilize the lattice oxygen redox, these strategies often lead to capacity sacrifice and lattice mismatch issues [16,17,18,19]. The lack of effective approaches for robust oxygen redox stabilization remains a longstanding bottleneck hindering the practical application of Li-rich layered oxide cathodes.

Drawing inspiration from the design of Na-O-M (where M = Li, a vacancy, or other elements) configurations in sodium-based layered oxides to enable controllable anionic oxygen redox [20,21,22], it is essential to devise analogous configurations in Li-rich layered oxides that simultaneously deliver additional capacity and stabilize lattice oxygen redox. Based on Pauling’s electrostatic valence rule [23,24], a series of novel structural configurations have been proposed to enrich the coordination environments and access usable oxygen redox activity in lithium-based oxides. For example, a Li-O-Na configuration can be introduced into Li[Na_1/3_Ru_2/3_]O_2_ via ion exchange from the Na precursor Na[Na_1/3_Ru_2/3_]O_2_. The presence of Na in the transition metal layer can be verified by ^23^Na solid-state nuclear magnetic resonance (NMR). The material delivers a first-cycle discharge capacity of 234 mAh g^−1^, exhibits nearly undetectable oxygen loss during the first cycle as monitored by differential electrochemical mass spectrometry (DEMS), and retains 82.9% of its capacity after 800 cycles [25]. Li-O-M (M = Sr, Mg, Al, Fe) configurations were successfully introduced into Li_1.187_Ni_0.136_Co_0.136_Mn_0.533_O_2_ via brine quenching. X-ray absorption fine structure (EXAFS) analysis confirmed the modified coordination environment of the transition metal atoms. DFT calculations revealed that the electronegativity of M^n+^ lowers the energy level of the unhybridized O 2p state, reduces TM-O’s covalency, and thereby stabilizes the lattice oxygen redox. The material delivers a first-cycle discharge capacity of 280 mAh g^−1^ and retains 80% of its capacity after 2000 cycles [26]. The evolution of atomic vacancies in Li_4/7_[□_1/7_Mn_6/7_]O_2_, prepared via ion exchange from the Na precursor Na_2_Mn_3_O_7_ in a molten salt environment, was monitored by ^7^Li solid-state NMR spectroscopy. The introduction of Li-O-vacancy configurations effectively suppressed lattice oxygen release. Reversible structural evolution and lithium migration were tracked by in situ electrochemical X-ray diffraction (XRD). The material delivers a first-cycle discharge capacity of 312 mAh g^−1^ and retains 80.7% of its capacity over 20 cycles at low current rates [27]. Neither brine quenching nor ion exchange methods are suitable for the large-scale production of lithium-rich layered oxides.

In this work, based on a straightforward composition-control approach, a Li-O-vacancy configuration coupled with a layered/spinel composite structure is introduced into Li-rich layered oxides, which not only activates oxygen redox to provide additional capacity but also stabilizes the layered framework. The composition of Ni, Co, and Mn is set as 4:1:5, with a Li/TM ratio of 0.95. The high Mn content is designed to promote Li’s incorporation into the transition metal (TM) layer. Using state-of-the-art integrated differential phase contrast scanning transmission electron microscopy (iDPC-STEM), Li and TM atoms are simultaneously visualized at atomic resolution. A high density of TM vacancies is observed within the TM layer, deviating from the ideal LiMn_6_ configuration and spontaneously forming a defective (Li/Mn)Mn_6−x_ arrangement. This localized short-range ordering introduces Li-O-V (V = vacancy) configurations, which are demonstrated to enable stable oxygen redox activity. Insufficient lithium content leads to extensive Ni migration into the Li layer, resulting in the formation of spinel-like domains in local regions. Benefiting from the strain relaxation provided by atomic vacancies and the structural stability of the layered/spinel heterostructure, the electrode exhibits reversible oxygen redox during initial cycling, delivering a high discharge capacity of 233 mAh·g^−1^ with a Coulombic efficiency of 90.5%. Owing to its enhanced structural and electrochemical stability, the material retains 86.5% of its capacity after 100 cycles.

## 2. Materials and Methods

### 2.1. Material Synthesis

The preparation route of the material adopts the high-temperature solid-phase synthesis process of mature carbonate precursors. According to the molar ratio of Ni, Co, and Mn (Ni:Co:Mn = 4:1:5) in the required precursor, NiSO_4_·6H_2_O, CoSO_4_·7H_2_O, and MnSO_4_·H_2_O were weighed to prepare a 2 mol L^−1^ sulfate solution. The temperature of the coprecipitation solution was fixed at 60 °C. Considering the balance of solubility, suppression of side reactions, and control of particle nucleation and growth, a pH of 8, commonly used in the synthesis process, was adopted. Then, a 2 mol L^−1^ sulfate mixed solution, a 2 mol L^−1^ Na_2_CO_3_ solution, and a 0.2 mol L^−1^ NH_4_OH solution were simultaneously pumped into the continuously stirred reactor with three peristaltic pumps at the same rate. The corresponding carbonate precursors were obtained by filtration, washing, and drying. The Li_2_CO_3_ and carbonate precursors were weighed according to the molar ratio of transition metal to Li (Li/TM = 0.95 and Li/TM = 1.1), mixed evenly, and then held at 500 °C for 5 h and at 850 °C for 12 h. Finally, Li_0.938_Ni_0.395_Co_0.099_Mn_0.494_O_2_ and Li_1.048_Ni_0.381_Co_0.095_Mn_0.476_O_2_ were obtained. Based on the Li/TM, these samples were simplified as LR-NCM415-0.95 and LR-NCM415-1.1, respectively.

### 2.2. Characterization

Long-range structural analysis of the as-prepared materials was carried out with in-house X-ray diffraction (XRD) using a Bruker D8 diffractometer (Cu-Ka radiation, Billerica, MA, USA). Inductively coupled plasma optical emission spectrometry (ICP-OES, SPECTRO ARCOS II, SPECTRO, Kleve, Germany) was used for the elemental analysis. A field emission scanning electron microscope (FSEM, Hitachi S-4800, Tokyo, Japan) was used to analyze the morphology of pristine materials. STEM-HAADF and STEM-iDPC were performed using a FEI Spectra 300 transmission electron microscope (Waltham, MA, USA) equipped with a double aberration corrector, operated at an accelerating voltage of 300 kV. The probe conditions were maintained at a convergence angle of 24.9 mrad and a beam current of 50 pA. All images were acquired with a resolution of 2048 × 2048 pixels and a dwell time of 10 μs.

### 2.3. Electrochemical Performance

The tests of electrochemical performance were conducted with CR2032-type coin cells. For this, 80 wt.% cathode powders, 10 wt.% carbon super P, and 10 wt.% polyvinylidene fluoride (PVDF) binder were homogenously mixed by hand-grinding with a mortar and pestle to prepare the electrodes. Circular cathode electrodes, a Celgard 2502 separator (Charlotte, NC, USA), and round lithium foil were assembled into coin cells in an argon-filled glove box associated with oxygen levels less than 0.1 ppm. The electrolyte consisted of ethylene carbonate–dimethyl carbonate (1:1 volume ratio) with LiPF_6_ (1.0 M). The galvanostatic charge–discharge testing was conducted in the potential range of 2.0–4.8 V vs. Li^+^/Li^0^.

## 3. Results

**Figure 1:**
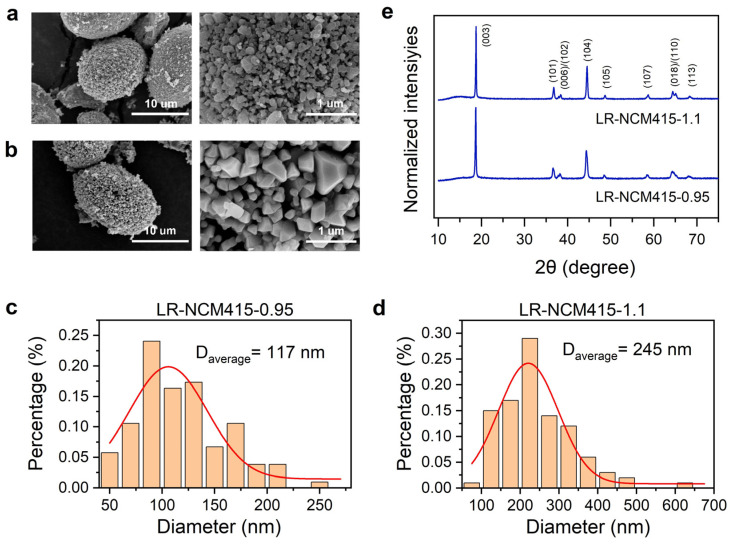
Particle morphology and crystal structure. The SEM results of the (**a**) LR-NCM415-0.95 and (**b**) LR-NCM415-1.1. Particle size distribution diagram of (**c**) LR-NCM415-0.95 and (**d**) LR-NCM415-1.1. The red line represents the fitting result of Gaussian function (**e**) Diffractograms of powdered cathode materials.

The Li-rich layered oxides LR-NCM415-0.95 and LR-NCM415-1.1 were synthesized via a high-temperature solid-state reaction using carbonate precursors [28,29]. Both samples were derived from the same precursor, with the numbers 0.95 and 1.1 denoting their respective Li/TM molar ratios. The specific molar ratios between the elements, as determined by inductively coupled plasma emission spectrometry (ICP-OES) analysis, are provided in Table S1. As shown in Figure 1a,b, LR-NCM415-0.95 and LR-NCM415-1.1 exhibit typical morphological features of layered oxide cathode materials, consisting of closely packed primary particles that aggregate into larger spherical secondary particles [30]. This microstructure not only facilitates full electrolyte wetting of the cathode’s surface but also enhances the electrode’s compacted density. Figure 1c,d present the secondary particle size distributions derived from SEM analysis. The particle sizes for both samples approximately follow a normal distribution. The average secondary particle diameters for LR-NCM415-0.95 and LR-NCM415-1.1 are 117 nm and 245 nm, respectively. Figure 1e presents the X-ray diffraction (XRD) patterns of LR-NCM415-0.95 and LR-NCM415-1.1. Both samples show main diffraction peaks corresponding to the α-NaFeO_2_ structure (space group R-3m) [31,32,33,34]. In contrast to typical Li-rich layered oxides, the superlattice reflections at 20–25°, which arise from short-range LiMn_6_ ordering between the Li and transition metal atoms, are nearly absent. The clear splitting of the (018)/(110) peaks represent a well-developed layered structure, with LR-NCM415-1.1 exhibiting a more pronounced splitting, corresponding to a better-ordered layered framework.

**Figure 2:**
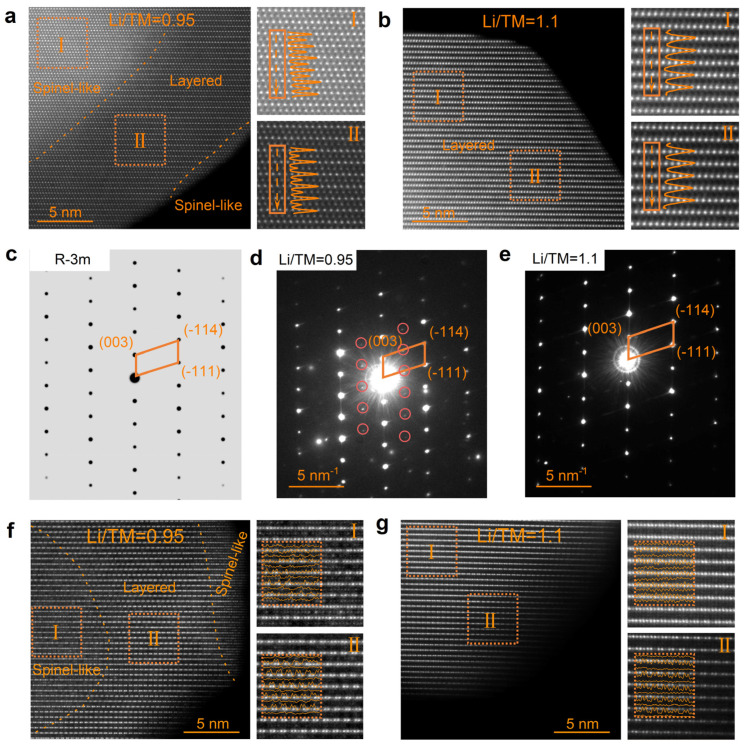
The STEM-HAADF results used to compare the structural integrity. The atomic configuration along the [110]_R_ zone axis of (**a**) LR-NCM415-0.95 and (**b**) LR-NCM415-1.1. SAED of (**c**) LiCoO_2_, (**d**) LR-NCM415-0.95, and (**e**) LR-NCM415-1.1. Atomic level structure along the [1–10]_M_ zone axis of (**f**) LR-NCM415-0.95 and (**g**) LR-NCM415-1.1.

To compare the atomic arrangement of the transition metal (TM), STEM-HAADF images of the as-prepared Li-rich layered oxides were acquired along the [110]_R_ zone axis in their pristine state (Figure 2a,b). Owing to the strong atomic-number (Z)-dependent contrast (∝Z^2^) in HAADF imaging, the TM columns appear as bright dots, while the Li and O columns are nearly invisible, providing an ideal means to monitor structural integrity [35]. As shown in Figure 2b, a characteristic alternating stacking of TM layers and Li layers is observed in LR-NCM415-1.1. For more detailed analysis, local magnifications were performed between the interior and the surface, marked as Regions I and II, respectively. The intensity line profile taken within the blue rectangular box confirms a well-defined layered arrangement, where the intensity peaks correspond to TM layers and the troughs correspond to Li layers. In contrast, the structure of LR-NCM415-0.95, synthesized with an insufficient lithium source, is more complex, as shown in Figure 2a. During sintering, the precursor undergoes an intermediate transformation involving spinel/rock-salt phases [36,37,38]. Due to the limited lithium supply and the slow kinetics of lithium-ion diffusion, the transformation is delayed within the interior, leaving residual spinel-like features. Unlike the well-defined layered structure of LR-NCM415-1.1, numerous bright spots are also visible within the Li layers in LR-NCM415-0.95. The intensity line profile extracted from the selected rectangular region shows continuous oscillations in the spinel-like area (Region I), and small peaks appear between the signals corresponding to the transition metal layers, indicating a partially disrupted layered arrangement (Region II). The evaporation of lithium salts during synthesis further results in a thin spinel-like surface layer. Because the DPC imaging signal arises directly from electron interactions, it exhibits high sensitivity to the domain distribution. As shown in Figure S3, the DPC data are largely consistent with the distribution of spinel domains identified by HAADF, which are primarily concentrated in the particles’ interior. Moreover, Raman spectroscopy images, as a short-range order characterization, were also acquired and are shown in Figure S4. The two main peaks around 490 cm^−1^ and 605 cm^−1^ are contributed by E_g_ and A_1g_ modes from the R-3m space group. The Raman peak at 655 cm^−1^ is indexed to the typical spinel structure of Li_4_Mn_5_O_12_. Figure 2e presents the selected area electron diffraction (SAED) pattern of LR-NCM415-1.1, which resembles the simulated pattern of LiCoO_2_ with the R-3m space group (Figure 2c), with the reciprocal space lattice consisting of a series of periodically arranged bright diffraction spots. In contrast, the SAED pattern of LR-NCM415-0.95 (Figure 2d) displays the main diffraction spots along with additional impurity reflections.

Figure 2g presents the STEM-HAADF image of LR-NCM415-1.1 acquired along the [1–10]_M_ zone axis. The dumbbell-like bright spots within the transition metal (TM) layers originate from the honeycomb-type LiMn_6_ ordering, which is a characteristic superstructure of Li-rich layered oxides [28,29]. Because the surface remains in prolonged contact with molten lithium salt during sintering, typical dumbbell-like spots associated with Li_2_MnO_3_-like structure (the line profile intensities show a dense and sharp bimodal characteristic) are found to be concentrated on the surface (Region II). In contrast, the interior region is dominated by the conventional layered oxide structure (LiTMO_2_). In comparison, the sample LR-NCM415-0.95 (Figure 2f) exhibits a sandwich-like structural distribution along the same zone axis, consistent with the observations along [110]_R_ in Figure 2a, where a defective layered structure appears in the middle region. Interestingly, despite the lower lithium content used during synthesis, dumbbell-like spots are much more pronounced in LR-NCM415-0.95.

**Figure 3:**
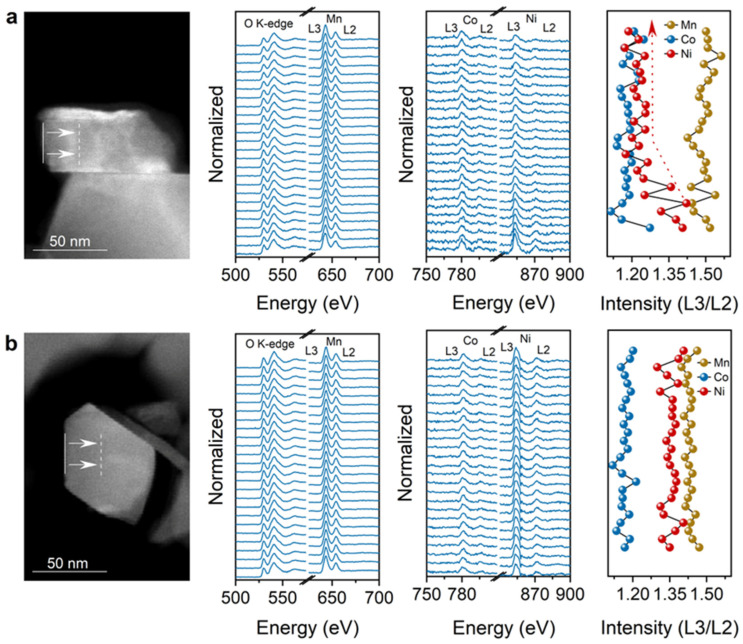
STEM-EELS of (**a**) LR-NCM415-0.95 and (**b**) LR-NCM415-1.1. The scans are composed of the O K edge and Mn, Co, and Ni L_2_/L_3_ edges.

Electron energy loss spectroscopy (EELS) was used to track chemical valence variations, as shown in Figure 3. Because the 2p orbitals of the transition metal are excited during inelastic scattering, resulting in two distinct absorption edges. Due to spin–orbit coupling, these edges split and form a “double peak” structure, collectively referred to as the L-edge. The L_3_-edge and L_2_-edge correspond to transitions from the 2p_3_/_2_ and 2p_1_/_2_ core levels to unoccupied d orbitals, respectively. The intensity ratio L_3_/L_2_ can be used for a qualitative analysis of valence state changes of the transition metals (Ni, Co, and Mn) in the layered oxide cathode [39,40]. In LR-NCM415-1.1, the valence states of all transition metals show no significant spatial variation (Figure 3b). By contrast, in the sample LR-NCM415-0.95, the Ni valence gradually decreases over a distance of several tens of nanometers from the surface toward the bulk (Figure 3a), which correlates with the transition from a layered to a spinel-like structure. By combining this observation with the structural evolution revealed by HAADF-STEM, it can be inferred that Ni is the dominant transition metal species occupying Li layer sites. The HAADF results indicate that the occupancy of transition metal atoms in the lithium layer primarily occurs in the particles’ interior. Combined with the evolution of the L_3_/L_2_ intensity ratio from the surface to the bulk, reflecting changes in the chemical coordination environment of Ni, it can be inferred that the transition metal atoms occupying lithium layer sites are predominantly Ni.

**Figure 4:**
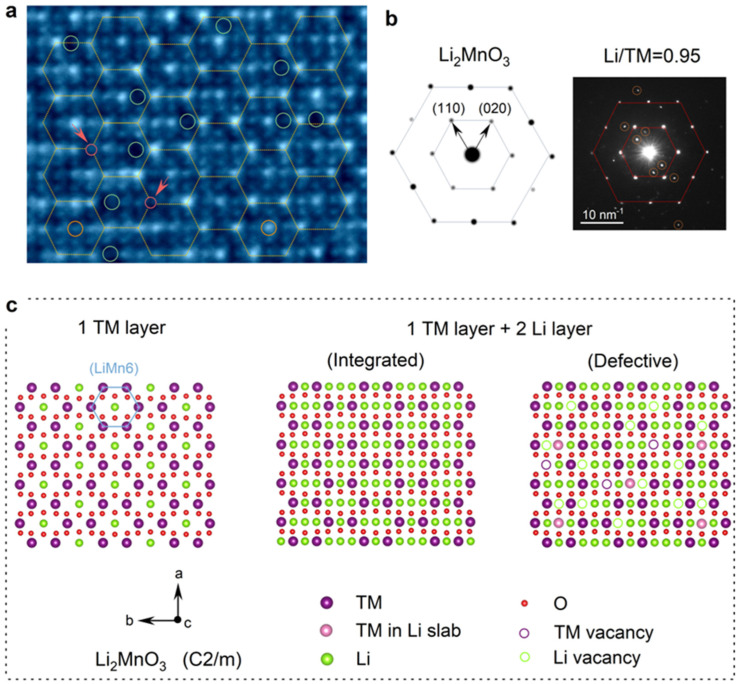
Short-range ordering within the transition metal layer. (**a**) iDPC-STEM results along [001]_M_ to monitor the integrities of the LiMn6. (**b**) Comparison of the SAED results between Li_2_MnO_3_ and LR-NCM415-0.95. (**c**) Structural schematic diagram depicting vacancy defects.

The state-of-the-art imaging technique iDPC-STEM significantly enhances the contrast of weakly scattering, low-atomic-number elements such as lithium and oxygen [41,42]. As shown in Figure 4a, the lattice structure of LR-NCM415-0.95, including TM and Li atomic columns, is clearly resolved along the [001]_M_ zone axis. Two kinds of atomic column projection models of the standard Li_2_MnO_3_ structure are considered in Figure 4c, one representing a single transition metal layer, and the other a single transition metal layer plus twin Li layers (the corresponding projection along the [1–10]_M_ zone axis with the same layer count is shown in Figure S5). In the short-range LiMn_6_ ordering, transition metal atoms occupy the six corners of a hexagon, and a Li atom occupies the center. Given that the real crystal consists of hundreds of atom layers along the electron beam’s direction, the atomic arrangement observed by STEM corresponds more closely to the projection of a single transition metal layer plus double Li layers. In Figure 4a, the brightest dots correspond to transition metal (TM) atoms, and the potential LiMn_6_ honeycomb units are outlined with hexagons. A significant number of atomic vacancies are observed at the hexagons’ corners (marked with red circles) and inside the hexagons (marked with green circles), representing TM vacancies and Li vacancies, respectively. Note that only the central position of the hexagon is considered to belong to the LiMn_6_ unit; thus, all green circles indicate vacancies within the Li layer. Furthermore, the central positions enclosed by yellow circles exhibit both bright and dark contrasts, corresponding to Li atoms and Mn atoms, respectively (see the coordination configurations in Figure S6). In summary, the absence of Mn atoms and their substitution into Li sites transforms the ideal LiMn_6_ configuration into a defective (Li/Mn)Mn_6−x_ structure. As shown in Figure 4b, compared with the simulated SAED pattern of standard Li_2_MnO_3_, the experimental diffraction exhibits the main spots arranged in a hexagonal pattern, accompanied by an additional set of symmetrical spots. These extra periodic reflections indicate that the (Li/Mn)Mn_6−x_ configuration retains a long-range periodicity.

**Figure 5:**
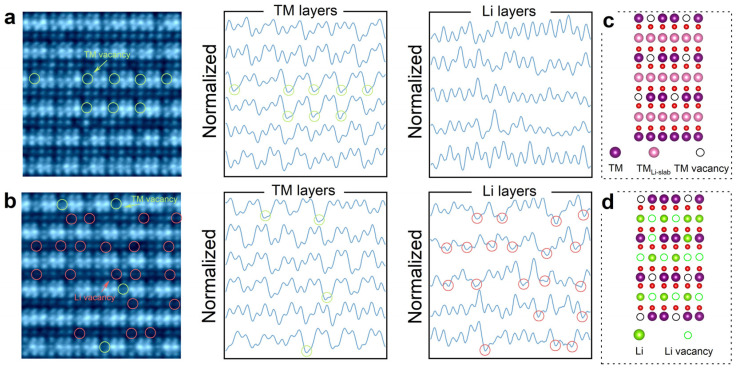
iDPC-STEM results used to visualize the defects’ distribution in LR-NCM415-0.95 along the [1–10]_M_ (**a**) within the interior and (**b**) on the surface. The line profile intensity along the TM layers and the Li layers is also placed on the right. Structure diagram of the (**c**) bulk and (**d**) surface. All the red spheres represent oxygen atoms.

The lattice structure of LR-NCM415-0.95, including the TM, O, and Li columns, is clearly resolved along the [1–10]_M_ zone axis in Figure 5a,b. The images in Figure 5a and Figure 5b correspond to the interior and the surface of the same particle, respectively. Significant differences are observed within the Li layers between these two regions. The image contrast of the lithium atomic layer is significantly higher in Figure 5a than in Figure 5b, indicating a greater occupancy of lithium atomic sites by transition metal atoms in the particles’ interior compared with the surface region. In addition, numerous missing atomic column projection signals (marked by red circles) are observed in Figure 5b, indicating the presence of abundant lithium atom vacancies on the particles’ surface. In addition, a series of vacancies were observed in the transition metal layers of both sampled regions, as indicated by the green circles. For a more intuitive comparison of atomic occupation, intensity profiles extracted along the TM layers and the Li layer across the full width of each image are displayed on the right. Based on the positions of the nodes (atomic sites) in the intensity profiles, a series of atomic vacancies corresponding to the atomic image can be identified. The disappearance of a peak signal within the atom layers are indicators of the vacancies (marked by green and red circles). On the basis of the [1–10]_M_ iDPC results, we infer that the dual-spot pattern exhibited in HAADF is not entirely that of the typical “TM-TM-Li” pattern but is mixed with that of “TM-TM-vacancy”. Table S2 presents the refined occupancy of Mn^4+^ in the transition metal layer, from which the corresponding vacancy fraction is estimated to be approximately 10.9%. On the basis of the structural analysis of the particles’ interior and surface, we propose the schematic model illustrated below. In the inner region (Figure 5c), the transition metal layer contains sparse metal vacancies, while the lithium layer is fully occupied by transition metal atoms. In the surface region (Figure 5d), vacancies are present in both the transition metal layer and the lithium layer. Additionally, all metal vacancies exhibit periodic arrangement.

**Figure 6:**
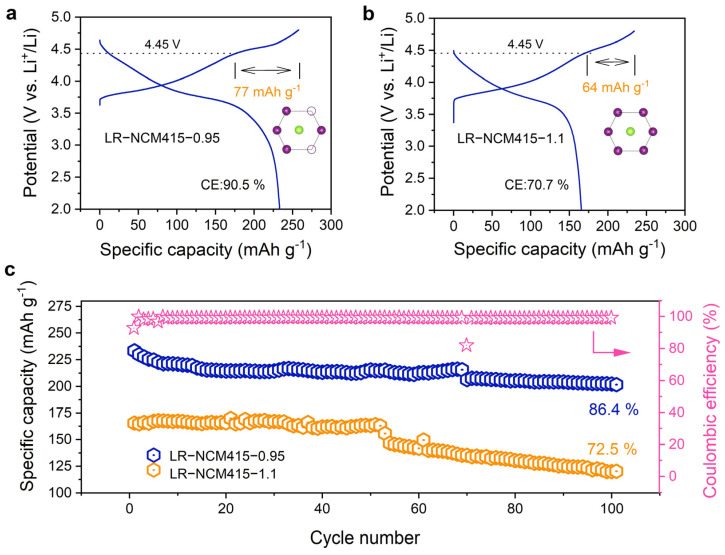
Comparison of the electrochemical performance in a coin cell vs. Li^+^/Li. Initial charge–discharge curves were compared between (**a**) LR-NCM415-0.95 and (**b**) LR-NCM415-1.1 under 0.1 C (2.0–4.8 V). (**c**) Long cycling performance.

Figure 6a,b present the initial charge–discharge curves. Both samples show a distinct voltage plateau during charging (4.45 V), which originates from lattice oxygen oxidation and represents a characteristic electrochemical signature of Li-rich layered oxides [43]. Notably, with a lower Li content, LR-NCM415-0.95 delivers a larger oxygen redox contribution, reaching 77 mAh·g^−1^. The respective charge capacities are 258 mAh·g^−1^ (LR-NCM415-0.95) and 234 mAh·g^−1^ (LR-NCM415-1.1), while the discharge capacities are 233 mAh·g^−1^ and 165 mAh·g^−1^. This results in an initial Coulombic efficiency decreasing from 90.5% for LR-NCM415-0.95 to 70.7% for LR-NCM415-1.1. The higher initial Coulombic efficiency and greater oxygen capacity contribution of LR-NCM415-0.95 demonstrate its effective utilization of oxygen redox activity. After 100 cycles at a rate of 0.1 C, the capacity retention reaches 86.4%, with the corresponding performance comparisons provided in Table S3. The Coulombic efficiency of LR-NCM415-0.95 over 100 cycles is also provided. It can be seen that the efficiency gradually increases from near 90% in the first cycle to nearly 100% and remains stable during cycling. The specific capacity as a function of cycle numbers at different C rates for LR-NCM415-0.95 is provided in Figure S8. When the current rate is restored to 0.1 C, the capacity recovers to its initial level, demonstrating excellent electrochemical stability. Figure S9 presents the particle morphology of the sample LR-NCM415-0.95 after 100 cycles. Compared with the pristine morphology in Figure 1, the particles’ surfaces appear rougher and more porous, attributed to corrosion by HF acid generated from LiPF_6_’s decomposition in the electrolyte. However, unlike the severe fracture of spherical secondary particles observed for the sample LR-NCM415-1.1 during the first cycle, LR-NCM415-0.95 retains good morphological integrity after 100 cycles. According to conventional understanding, the Li-O-Li configuration associated with local LiMn_6_ ordering enables charge compensation through non-hybridized O 2p states. Our experimental results reveal that the Li-O-V (V = vacancy) configuration can likewise activate lattice oxygen redox.

## 4. Discussion

Li-rich layered oxide cathodes, recognized as derivatives of Li_2_MnO_3_, are widely accepted to form a nanocomposite consisting of the layered phase LiTMO_2_ (Li^+^ resides exclusively in the Li^+^ layer) and the Li-rich phase Li_2_MnO_3_ (Li^+^ partially occupies the transition metal layer sites) [44]. Given this kind of structure, the transition metals (Ni and Co) in the LiTMO_2_ domain contribute cationic capacity, while the lattice oxygen in the Li_2_MnO_3_ domain provides anionic capacity. Notably, unlike conventional NCM layered cathodes with a near-stoichiometric Li/TM ratio close to 1, the addition of excess lithium salt (Li/TM > 1) during the sintering stage is considered to be essential for the formation of nanoscale Li-rich phases. However, in this work, given the relatively high Mn content (uncommon in traditional NCM layered oxides), iDPC-STEM characterization of (Li/Mn)Mn_6−x_ revealed that even with a Li-deficient environment (Li/TM = 0.95), Li atoms preferentially enter the transition metal layer and coordinate with Mn atoms.

To maintain structural stability and charge balance, a significant amount of Ni migrates into the lithium layer, providing structural support. The distribution of Mn in the transition metal layer has also been reconfigured, with a fraction of Mn occupying sites that would be filled by Li atoms in an ideal lithium-rich phase, while the remaining Mn participates in forming short-range ordered defect superlattice. To achieve the lowest energy state, Mn vacancies and the Li spontaneously arrange into a short-range (Li/Mn)Mn_6−x_ ordering structure. The observed lattice oxygen capacity of 77 mAh·g^−1^ in the first charge curve confirms that (Li/Mn)Mn_6−x_ can still activate substantial lattice oxygen redox activity. The excellent Coulombic efficiency exhibited by LR-NCM415-0.95 can be attributed to two main factors. Firstly, the mixed layered–spinel structure provides structural support during Li^+^ extraction. X-ray diffraction patterns collected at selected states (pristine, charged to 4.4 V, charged to 4.8 V, and discharged to 2.0 V, as shown in Figure S10) demonstrate the structural stability of LR-NCM415-0.95. The shift in the (003) diffraction peak position is much smaller for LR-NCM415-0.95 than for LR-NCM415-1.1, indicating smaller unit-cell volume changes and better structural integrity in LR-NCM415-0.95 throughout electrochemical cycling. Secondly, the abundant atomic vacancies can buffer volume changes during cycling: During the electrochemical insertion and extraction of lithium ions, the repulsive force between adjacent oxygen layers undergoes significant changes, resulting in substantial expansion and contraction of the unit cell’s volume. This volume variation not only compromises the morphological integrity of individual particles but also induces interparticle stress, leading to particle fracture. The spherical secondary particles in the sample LR-NCM415-0.95, composed of aggregated primary particles, maintained excellent structural integrity throughout the first charge–discharge cycle (shown in Figure S11a–c). Under deep de-lithiation conditions, the spherical secondary particles fractured irreversibly due to interparticle compression (shown in Figure S11e). Upon close inspection, the surfaces of the primary particles exhibited multiple cracks, which remained visible even after discharging to 2.0 V (shown in Figure S11f). The lower accumulated strain in LR-NCM415-0.95 allows the particles to maintain morphological integrity, thereby reducing surface/interface side reactions. When the redox process of lattice oxygen becomes uncontrolled, the fracture of TM-O bonds leads to interlayer slipping and the rapid degradation of the layered structure. A comparison of atomic structures after charging to 4.8 V reveals that LR-NCM415-0.95 retains a highly ordered structural configuration compared with its initial state, maintaining an overall cubic close-packed arrangement (shown in Figure S12a). The particles’ interior exhibits a spinel-like phase, while the surface displays a defect-modified layered structure. In contrast, LR-NCM415-1.1 shows notable lattice distortion due to accumulated stress and strain (Figure S12b). Geometric phase analysis (Figure S12c,d) further confirms a higher concentration of internal strain within the lattice of LR-NCM415-1.1 [28,45].

Cyclic voltammetry (CV) curves are presented in Figure S13. The oxidation peaks at approximately 4.0 V and 4.6 V correspond to the oxidation of Co^3+^/Ni^2+^ and O^2−^, respectively [46]. In LR-NCM415-0.95, the modified coordination environment of Ni alters the charge transfer kinetics, resulting in sharper transition metal oxidation peaks. The enhanced oxygen-related oxidation signals confirm that the defective (Li/Mn)Mn_6−x_ structure effectively activates higher oxygen redox capability. Moreover, LR-NCM415-0.95 delivers increased low-voltage discharge capacity, which can be ascribed to the incorporation of spinel-like structural features.

## 5. Conclusions

By increasing the Ni/Mn content and reducing the Li content, a defective short-range ordered (Li/Mn)Mn_6−x_ configuration is successfully constructed within the transition metal layer. The associated Li-O-V (V = vacancy) configuration is shown to enable efficient oxygen redox activity. Owing to the combined effects of Li deficiency and high Ni content, a mixed structure consisting of layered and spinel-like phases is obtained. Benefiting from the strain relaxation provided by vacancy clusters and the structural support from the spinel-like phase, the resulting LR-NCM415-0.95 cathode exhibits high Coulombic efficiency and stable cycling performance. This work offers an important structural design strategy for Li-rich layered oxide cathodes.

## Data Availability

The original contributions presented in this study are included in the article/Supplementary Materials. Further inquiries can be directed to the corresponding authors.

## Supplementary Materials

The following supporting information can be downloaded at https://www.mdpi.com/article/10.3390/ma19061240/s1, Figure S1. Energy dispersive X-ray spectroscopy (EDX) results. The distribution of Ni, Co, and Mn of (a) LR-NCM415-0.95 and (b) LR-NCM415-1.1. (c) The normalized EDX emission spectrometry and the quantitative results of element content. Figure S2. SEM results of LR-NCM415-0.95 samples obtained at different sintering temperatures: (a,b) 700 °C; (c,d) 750 °C; (e,f) 800 °C; (g,h) 850 °C; (i,j) 900 °C; (k,l) 950 °C. Figure S3. Differential phase contrast results of LR-NCM415-0.95. Figure S4. Raman patterns of the sample LR-NCM415-0.95. Figure S5. The atomic structure diagram of Li_2_MnO_3_ along the [1–10]_M_ zone axis. Figure S6. Hexagonal ring configuration in single-layer transition metal layer. (a) The center is Li; (b) the center is TM. Figure S7. Rietveld refinement pattern of (a) LR-NCM415-0.95 and (b) LR-NCM415-1.1. Figure S8. The rate performance of LR-NCM415-0.95. Figure S9. The SEM results of the LR-NCM415-0.95 after 100 cycling. Figure S10. Comparison of the charged XRD pattern. (a) LR-NCM415-0.95; (b) LR-NCM415-1.1. Figure S11. Comparison of SEM results under different cut-off voltages. SEM results of LR- NCM415-0.95 of (a) Charged to 4.4 V, (b) Charged to 4.8 V and (c) Discharegd to 2.0 V; SEM results of LR-NCM415-1.1 of (a) Charged to 4.4 V, (b) Charged to 4.8 V and (c) Discharegd to 2.0. Figure S12. Fully charged state structur comparison. HAADF-STEM results of (a) Sample LR-NCM415-0.95 and (b) Sample LR-NCM415-1.1; GPA results of (c) Sample LR-NCM415-0.95 and (d) LR-NCM415-1.1; (e) Structure diagram in fully charged state. Figure S13. CV curves of the LR-NCM415-0.95 and LR-NCM415-1.1. Table S1: ICP-OES results of elemental contents of Li, Ni, Co and Mn in LR-NCM415 with different lithium contents, Table S2: The refined atomic parameters of Mn^4+^ in Li_2_MnO_3_ component, Table S3: Comparison of electrochemical performance.
