# Supplementary material for: Constructing Li-O-Vacancy Configuration Coupling with a Layered/Spinel Mixed Structure in Li-Deficient Li-Rich Layered Oxides to Realize Stable Oxygen Redox
Source: Materials (Basel). 2026 Mar 21;19(6):1240. doi: 10.3390/ma19061240 (PMC13027962; doi:10.3390/ma19061240)
Supplement: Supplementary file 1 [file materials-19-01240-s001.zip › materials-4190109-supplementary.pdf]

Supporting Information

Constructing Li-O-Vacancy configuration coupling with layered/spinel mixed structure in Li-deficient Li-rich layered oxides to realize stable oxygen redox

Yibin Zhang[1, 2,+], Meng Wang[1,+], Bao Qiu[1, 2 *], Zhaoping Liu[1, 2*]

1. Ningbo Institute of Materials Technology & Engineering (NIMTE), Chinese Academy of Sciences, Ningbo 315201, P. R. China
2. Center of Materials Science and Optoelectronics Engineering, University of Chinese Academy of Sciences (UCAS), Beijing 100049, P. R. China

+These authors contributed equally

Corresponding authors

qiubao@nimte.ac.cn, liuzp@nimte.ac.cn

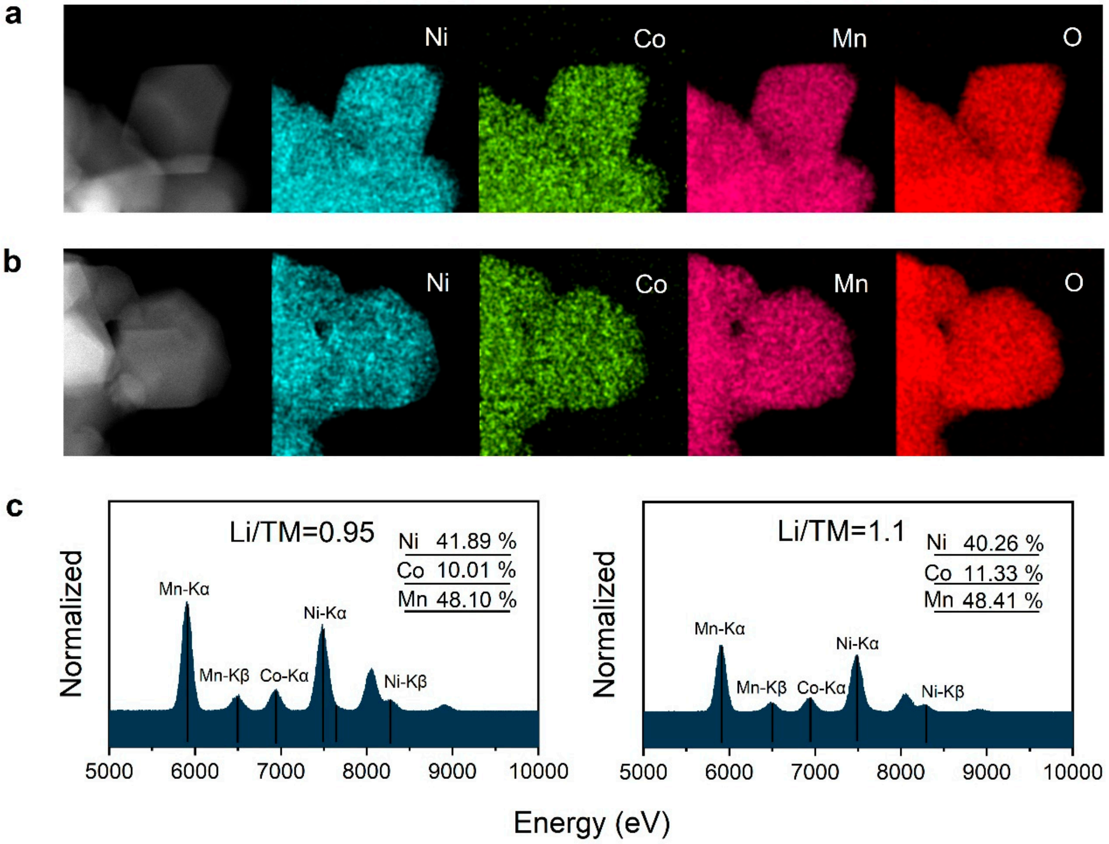

Figure S1 Energy Dispersive X-ray Spectroscopy (EDX) results. The distribution of Ni, Co and Mn of (a) LR-NCM415-0.95 and (b) LR-NCM415-1.1; (c) The normalized EDX emission spectrometry and the quantitative results of element content.

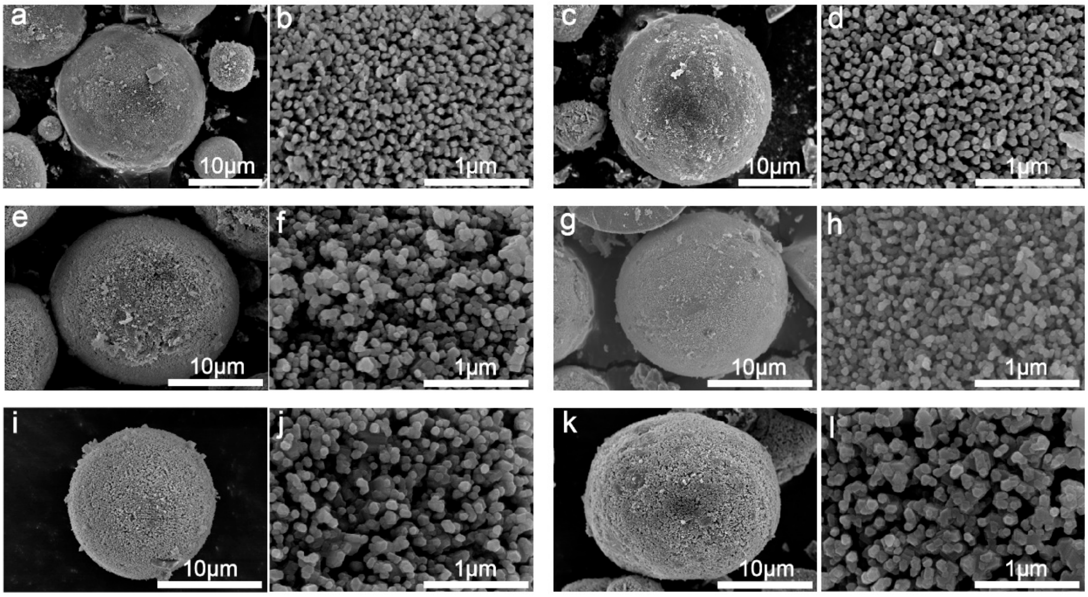

Figure S2. SEM results of LR-NCM415-0.95 samples obtained at different sintering temperatures. (a)-(b) 700 ℃; (c-d) 750 ℃; (e)-(f) 800 ℃; (g)-(h) 850 ℃; (i)-(j) 900 ℃; (k)-(l) 950 ℃.

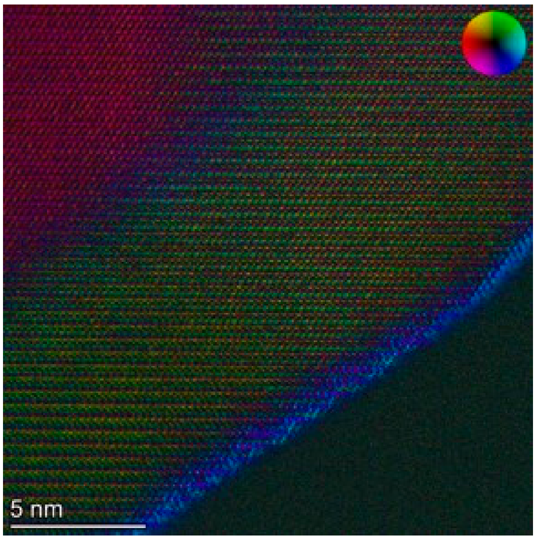

Figure S3. Differential phase contrast results of LR-NCM415-0.95.

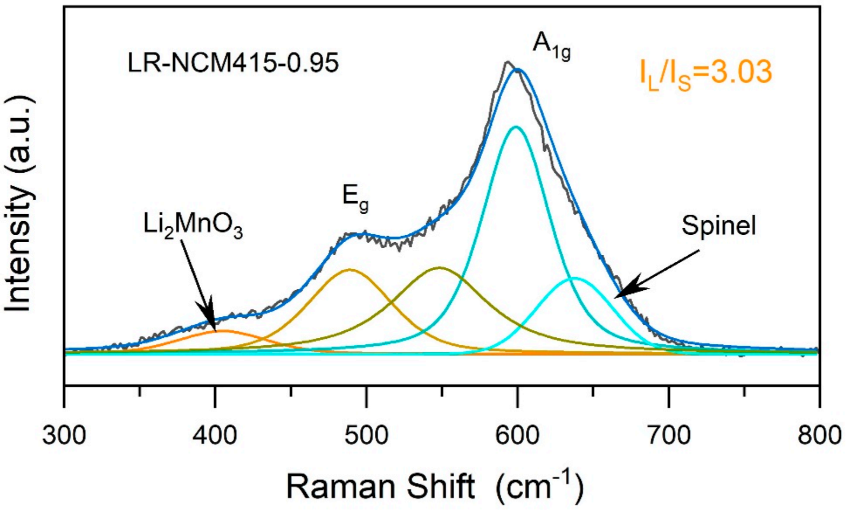

Figure S4. Raman patterns of sample LR-NCM415-0.95.

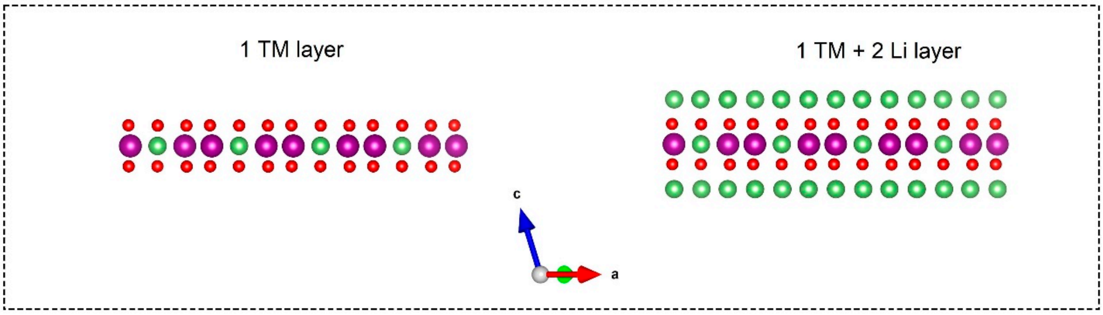

Figure S5. The atomic structure diagram of Li_2MnO_3 along the $[1\text{-}10]_M$ zone axis.

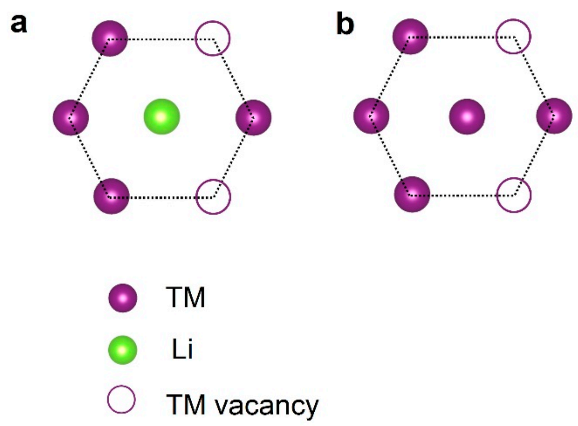

Figure S6. Hexagonal ring configuration in single-layer transition metal layer. (a) The center is Li; (b) The center is TM.

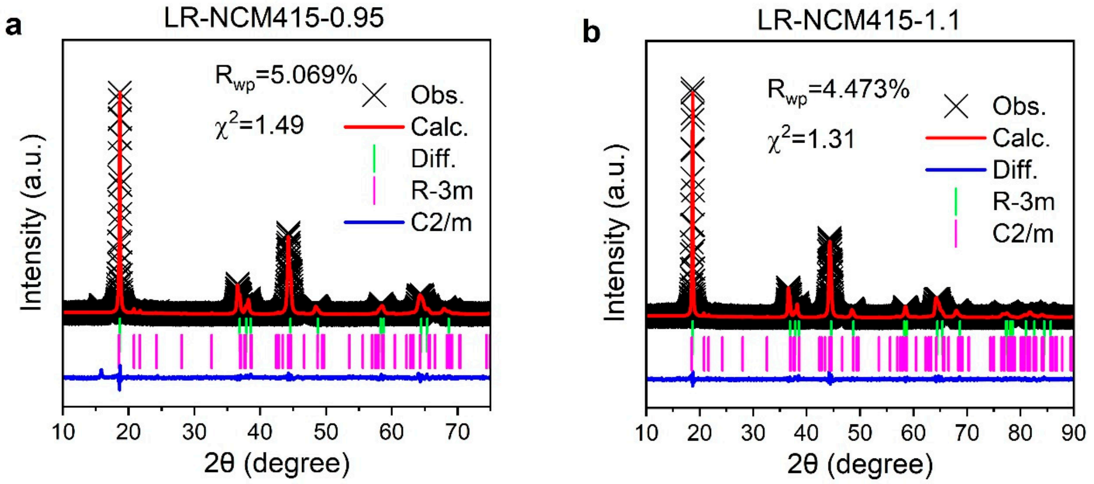

Figure S7. Rietveld refinement pattern of (a) LR-NCM415-0.95 and (b) LR-NCM415-1.1.

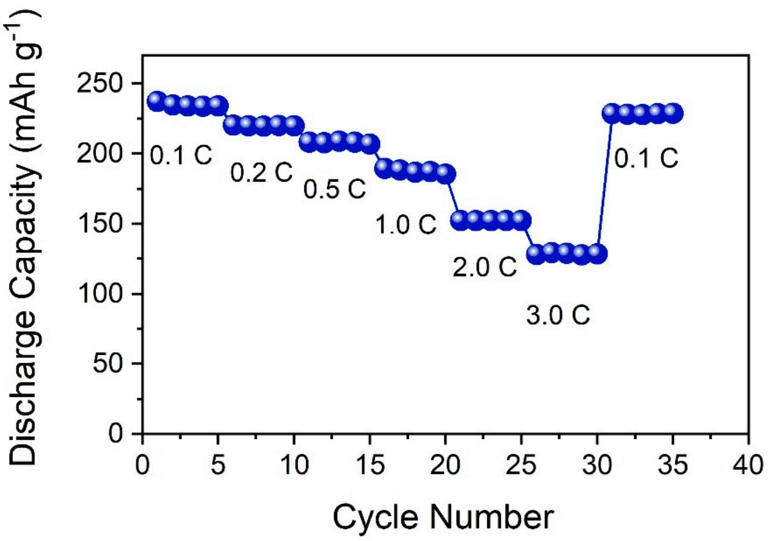

Figure S8. The rate performance of LR-NCM415-0.95

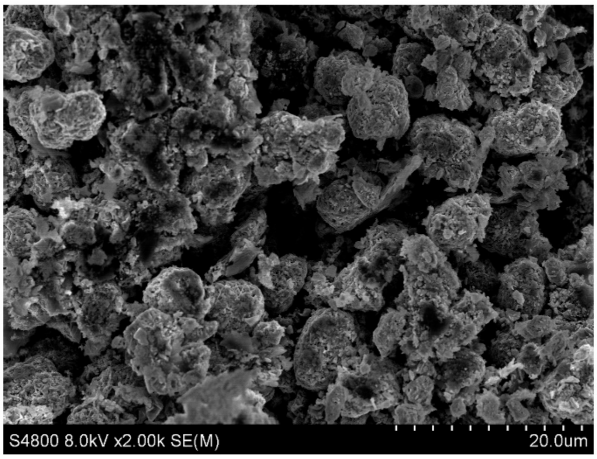

Figure S9. The SEM results of the LR-NCM415-0.95 after 100 cycling.

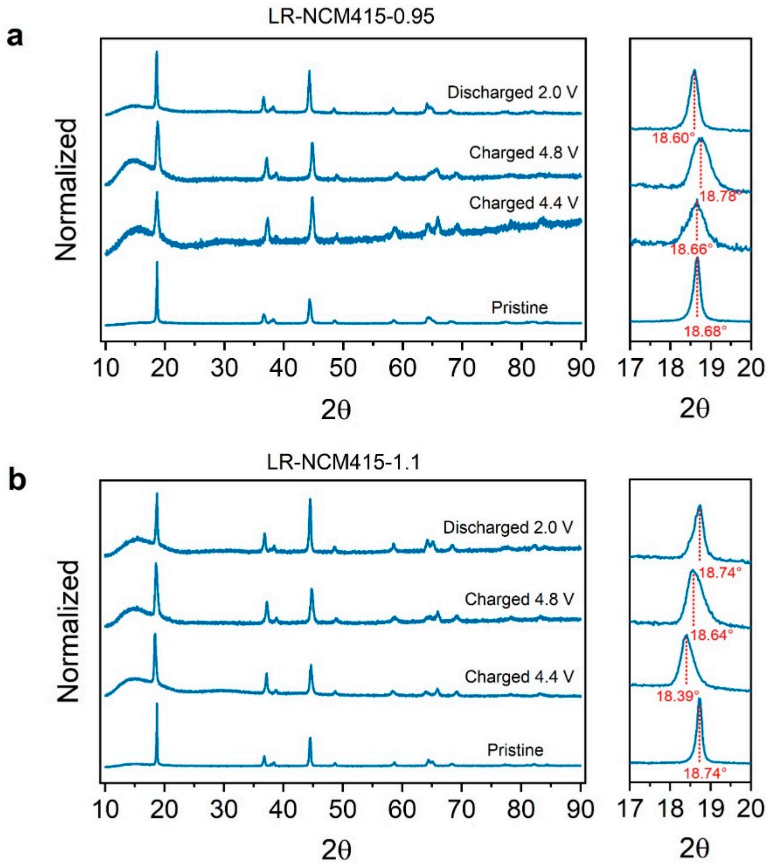

Figure S10. Comparison of the charged XRD pattern. (a) LR-NCM415-0.95; (b) LR-NCM415-1.1

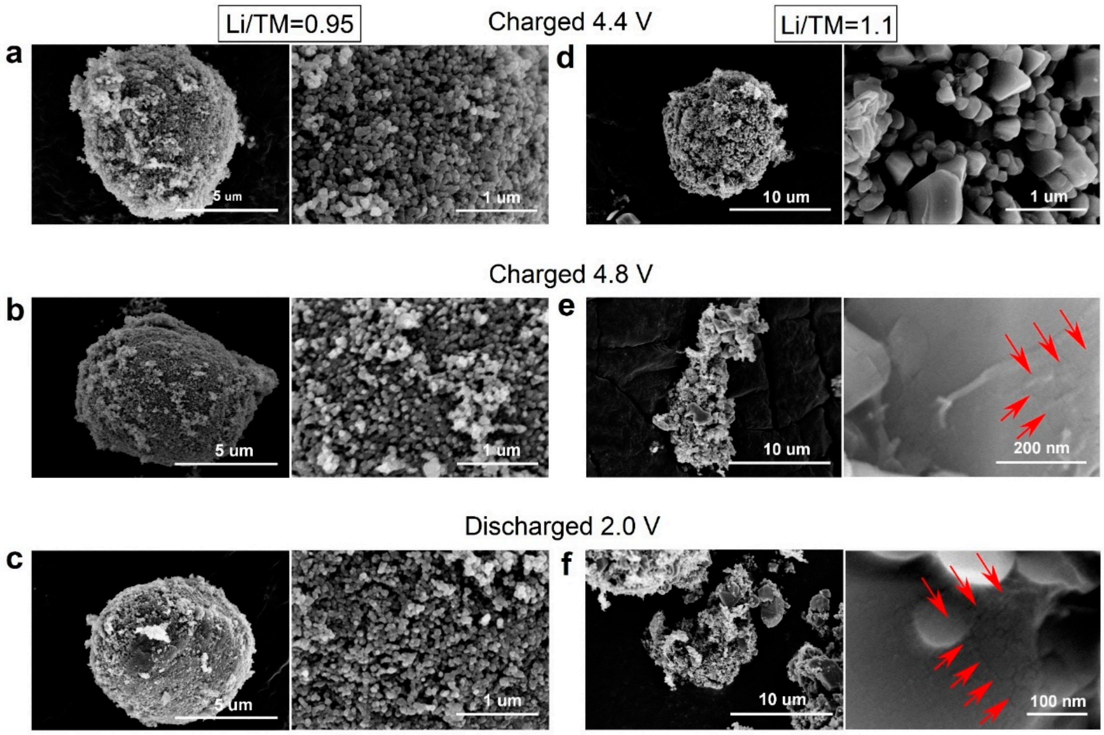

Figure S11. Comparison of SEM results under different cut-off voltages. SEM results of LR- NCM415-0.95 of (a) Charged to 4.4 V, (b) Charged to 4.8 V and (c) Discharegd to 2.0 V; SEM results of LR-NCM415-1.1 of (a) Charged to 4.4 V, (b) Charged to 4.8 V and (c) Discharegd to 2.0

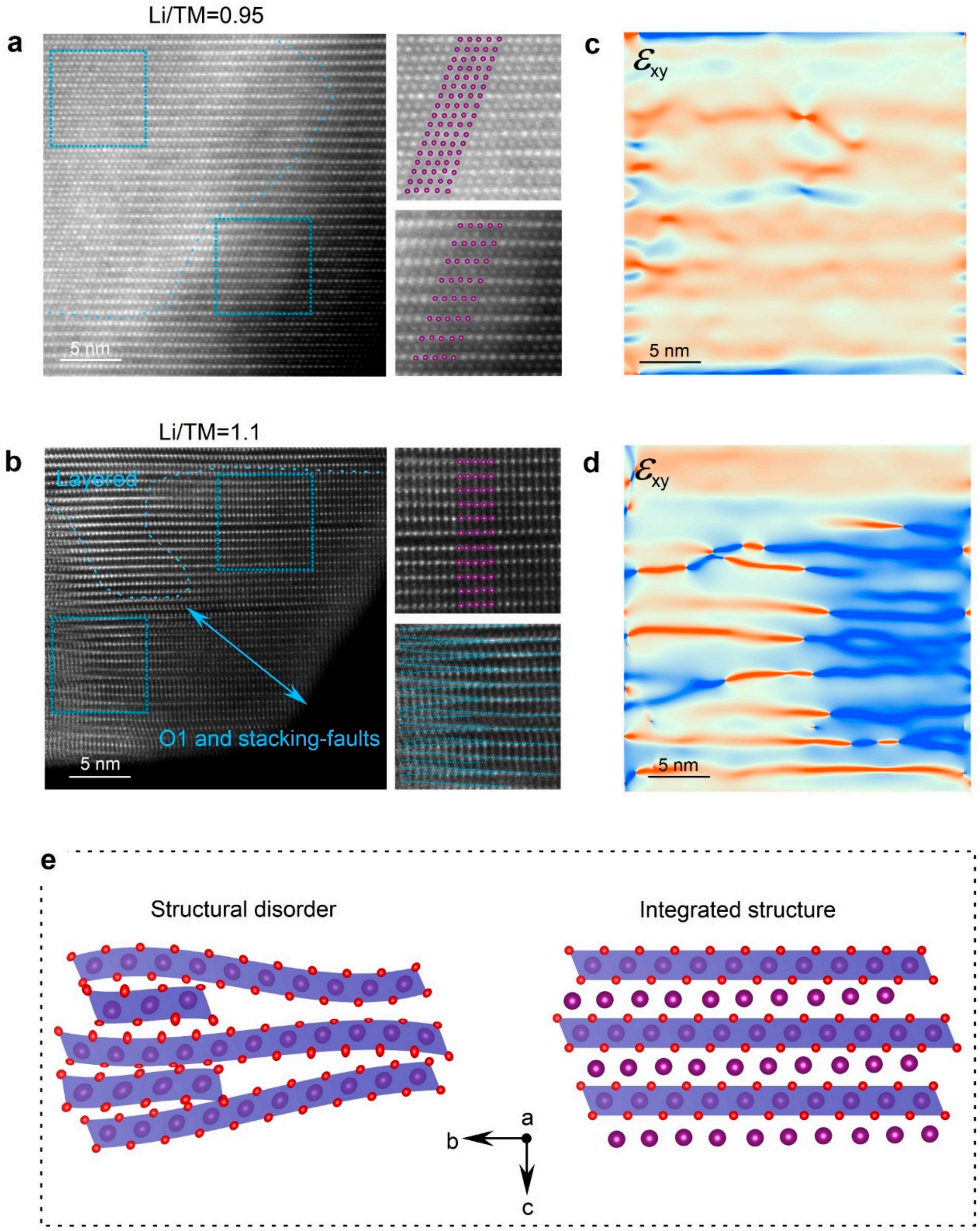

Figure S12. Fully charged state structur comparison. HAADF-STEM results of (a) Sample LR-NCM415-0.95 and (b) Sample LR-NCM415-1.1; GPA results of (c) Sample LR-NCM415-0.95 and (d) LR-NCM415-1.1; (e) Structure diagram in fully charged state

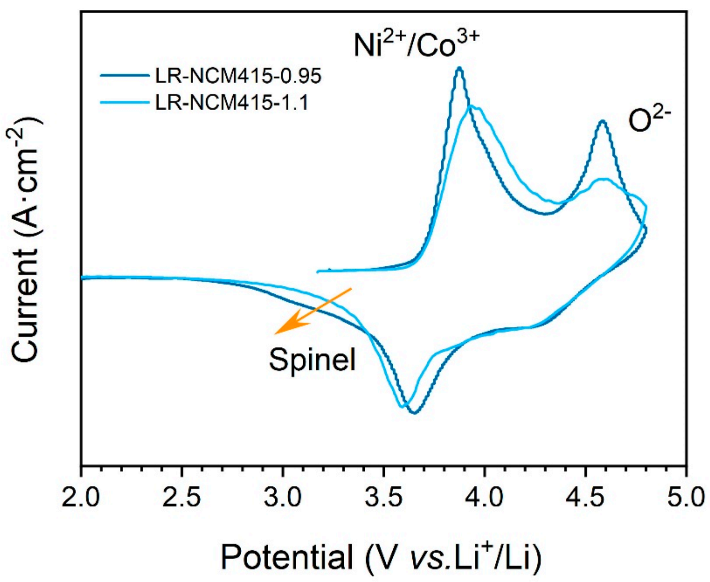

Figure S13. CV curves of the LR-NCM415-0.95 and LR-NCM415-1.1.

Table S1. ICP-OES results of elemental contents of Li, Ni, Co and Mn in LR-NCM415 with different lithium contents

**Sample**	**mole ratio**			
	**Li**	**Ni**	**Co**	**Mn**
Li/TM=0.95	0.963	0.401	0.105	0.494
Li/TM=1.10	1.093	0.402	0.105	0.493

Table S2. The refined atomic parameters of Mn^{4+} in Li_2MnO_3 component.

Li/TM	Atom	x	y	z	Frac.	Site sym.	Uiso
0.95	Mn^{4+}	0.00000	0.16627	0.00000	0.91563	2(y)	0.00773
1.1	Mn^{4+}	0.00000	0.16627	0.00000	1.00000	2(y)	0.00562

Table S3. Comparison of electrochemical performance.

Composition	Strategies	Initial capacity	Capacity retention	Industrialization	Ref.
$Li[Na_{1/3}Ru_{2/3}]O_2$	ion exchange	234 mAh g^{-1}	82.9 % (800 cycling)	Difficult	25
$Li_{1.187}Ni_{0.136}Co_{0.136}Mn_{0.533}O_2$	brine quench	280 mAh g^{-1}	80 % (2000 cycling)	Difficult	26
$Li_{4/7}[\square_{1/7}Mn_{6/7}]O_2$	ion exchange	312 mAh g^{-1}	80.7 % (20 cycling)	Difficult	27
$Li_{0.938}Ni_{0.395}Co_{0.099}Mn_{0.494}O_2$	Li/TM=0.95	233 mAh g^{1}	86.4 % (100 cycling)	Easy	This work
